# Do Dietitians Recommend Too Much Carbohydrate? A Cross‐Sectional Survey of Gestational Diabetes Mellitus Dietary Advice: A Patient Perspective

**DOI:** 10.1111/ajo.70121

**Published:** 2026-04-06

**Authors:** Laura C. Kourloufas, Robyn A. Barnes, Jeff R. Flack, Tang Wong

**Affiliations:** ^1^ Diabetes Centre, Bankstown‐Lidcombe Hospital Sydney Australia; ^2^ District Diabetes Coordinator, South Western Sydney Local Health District Sydney Australia; ^3^ School of Medicine, Western Sydney University Campbelltown New South Wales Australia; ^4^ Faculty of Medicine and Health, University of NSW Randwick New South Wales Australia

**Keywords:** carbohydrates, diabetes gestational, diet therapy, dietitians, pregnancy

## Abstract

**Background:**

Medical nutrition therapy is first‐line treatment for Gestational Diabetes Mellitus (GDM). The high popularity of low carbohydrate diets may impact on the acceptability of the diet recommended by dietitians for management of GDM.

**Aims:**

This study assessed the experience of women prescribed the Standardised Gestational Diabetes (SGD) diet (45%–50%, 25%–30%, 15%–20% from carbohydrate, fat and protein, respectively).

**Materials and Methods:**

A cross‐sectional survey of women with GDM was undertaken to collect viewpoints on the SGD diet. Pregnant women with pre‐gestational diabetes, twins, non‐English speakers and women with no or limited English literacy were excluded. Standard care was provided to all participants. The survey was offered at least one week after an individual dietitian review appointment.

**Results:**

A total of 135 women completed the survey. The majority perceived the SGD diet carbohydrate amounts and distribution as ‘about right’—79.3% (*n* = 107) and 72.6% (*n* = 98), respectively. Only 3.7% (*n* = 5) perceived the advised carbohydrate amount as ‘too much’ or ‘far too much’. Conversely, a considerable proportion reported that they were advised to reduce their intake and to increase meal‐snack frequency compared to their usual diet—62.9% (*n* = 85) and 43.7% (*n* = 59), respectively. Women reported high rates of diet compliance, with 70.4% (*n* = 95) ‘often’ or ‘always’ following the diet, despite 48.2% (*n* = 65) reporting the diet being ‘challenging’ or ‘very challenging’ to follow.

**Conclusions:**

Despite the popularity of low carbohydrate diets in the general population, our cohort of women with GDM perceived the amount and frequency of carbohydrate intake recommended by dietitians as ‘about right’.

## 1 Introduction

Gestational Diabetes Mellitus (GDM) is defined as ‘glucose intolerance of variable severity with onset or first recognition during pregnancy’ [1]. GDM affects approximately 12.4% of pregnancies within Australia [2], with evidence of higher incidence rates within Southeast Asian, South Asian and Middle Eastern ethnicities [2, 3, 4]. GDM has been associated with both short and long‐term adverse health outcomes for mothers and their infants [5].

Medical nutrition therapy (MNT) is widely accepted as first line treatment for GDM. MNT has been shown to improve fasting and postprandial blood glucose levels (BGLs) [6], reduce total pregnancy weight gain [7] reduce the need for medical therapy, and optimise neonatal outcomes [6]. Dietary carbohydrate amounts and distribution have historically been the focus of GDM MNT due to the impact on postprandial BGL excursions [8]. The Institute of Medicine (IOM) suggests a minimum of 175 g/day of carbohydrates for pregnant women [9]. According to a recent survey of Australian dietitians (*n* = 152), most provide GDM MNT aiming for macronutrient targets consistent with a high carbohydrate (> 45% energy), moderate protein (15%–25% energy) and moderate fat (15%–30% energy) diet [10].

Given MNT is first‐line therapy for GDM management, it would be of value to obtain the perspectives of the GDM diet from women living with GDM. We also wanted to assess whether the high general population interest in low carbohydrate diets impacts on the acceptability of the GDM diet, hereafter referred to as the Standardised Gestational Diabetes (SGD) diet [11].

To our knowledge this is the first study to collect women's perspectives on the SGD diet which is recommended at *Bankstown‐Lidcombe Hospital Diabetes Centre, location removed for peer review*, used across many diabetes services across Australia [10], and is in line with international guidelines including the Academy of Nutrition and Dietetics Gestational Diabetes Evidence‐Based Nutrition Practice Guidelines [9].

The study aimed to assess the patient experience of the SGD diet, in particular, their perspectives of the carbohydrate quantity and distribution recommended.

## 2 Materials and Methods

### 2.1 Study Design and Participants

A cross‐sectional survey of women with GDM managed by our department was administered from July 2017 to July 2019. Our Diabetes Centre is situated in a low‐socioeconomic status (SES), culturally diverse metropolitan area, with significant proportions of Middle Eastern, South Asian and Southeast Asian residents. The World Health Organisation diagnostic criteria were applied. Using a 75 g oral glucose tolerance test, one (or more) abnormal values: ≥ 5.1 mmol/L fasting, ≥ 10.0 mmol/L at 1 h or ≥ 8.5 mmol/L at 2 h were diagnostic of GDM [12]. Inclusion criteria were singleton pregnancies of English speaking and English literate women with GDM. Exclusion criteria were pregnant women with pre‐gestational diabetes, multiple pregnancies, non‐English speaking women requiring an interpreter and women with limited English literacy as measured by the Single Item Literacy Screener (SILS) [13]. The study was approved by the Human Research Ethics Committee of South Western Sydney Area Health Service (2019/ETH00064).

### 2.2 GDM Management and the SGD Diet

Standardised care was provided to all women within a multidisciplinary GDM clinic. Diabetes educators and dietitians provided initial group education and ongoing individualised review appointments. The diabetes educators taught self‐monitoring of blood glucose (SMBG) levels and explained treatment targets of < 5.3 mmol/L fasting, < 7.4 mmol/L at 1 h postprandial or < 7.0 mmol/L at 2 h postprandial. Dietitian initial group education included the following: healthy balanced eating for pregnancy, carbohydrate versus non‐carbohydrate, carbohydrate role, quantity, distribution and type; glycaemic index and guidelines for concentrated sources of sugar and fats. The SGD diet aimed for 45%–50% total energy as carbohydrate, 20%–25% as protein and 25%–30% as fat [10]. The total amount of carbohydrate advised was based on the US Dietary Reference Intake for pregnant women of a minimum of 175 g/day [9]. Women were advised to distribute carbohydrate over three meals and three snacks as follows: 30–45 g at breakfast, 45–60 g at lunch and dinner and 15–30 g at snacks [10]. In most cases, carbohydrate amounts were taught using household measures, rather than 15‐g carbohydrate exchanges.

At the initial session, culturally appropriate meal plans (Western, Middle Eastern, Asian or Indian cuisine) were provided to all women. These meal plans illustrated recommended meal and snack options. The Australian dietary guidelines for healthy eating in pregnancy were used to promote pregnancy nutritional adequacy [14].

All women in this study received an individual dietitian review appointment approximately 1 week after the initial group education, where individualised dietary advice was provided after review of their 5‐day food diary and SMBG results. This advice considered personal and cultural food preferences. The survey was offered during subsequent appointments within the GDM clinic service.

### 2.3 Survey Design

As a literature review confirmed a lack of validated tools to assessing SGD perception, a survey was developed and piloted. The final survey comprised 10 five‐point Likert scale questions with an additional ‘unsure’ response option, four open ended questions, one checkbox question and seven demographic data questions (see Supporting Information). The Likert scale questions explored patient perspectives of the SGD diet carbohydrate amounts, carbohydrate distribution and comparison to usual dietary intake, as well as SGD diet understanding, usefulness, practicality, compliance, ease of implementation and hunger levels experienced. The open‐ended questions captured participant food item preferences, dietary changes following SGD education, barriers to SGD diet compliance and general suggestions to improve the dietitian sessions. The checkbox question established carbohydrate knowledge and identification skills. De‐identified demographic data collected included: participant age, weeks' gestation at time of questionnaire completion, education, ethnicity, pre‐pregnancy weight, current weight and height.

### 2.4 Data Collection

The dietitian invited eligible women to participate in the survey whilst waiting to see other diabetes team members (usually Endocrinologist or Diabetes Educator). Women interested in receiving more information were provided a study information sheet, a survey and the SILS English Literacy Test. A SILS score of ‘4‐Often’ or ‘5‐Always’ indicated the participant required assistance reading English material and they were therefore excluded from the study. Anonymity was ensured by participants placing completed anonymised surveys into an envelope which they then placed in a closed drop‐box. Providing a completed questionnaire was taken as implied consent.

### 2.5 Data Analysis

Survey data were entered into Microsoft Excel and SPSS (version 24) for analysis. Descriptive statistics were generated as frequency of total number of responses for each question (%) and mean ± standard deviation (SD). Continuous and categorical data were analysed using independent samples *t*‐test or Chi‐squared test, respectively, with statistical significance *p* < 0.05. Qualitative data from survey open ended questions were grouped into same response types and analysed quantitatively.

## 3 Results

**FIGURE 1:**
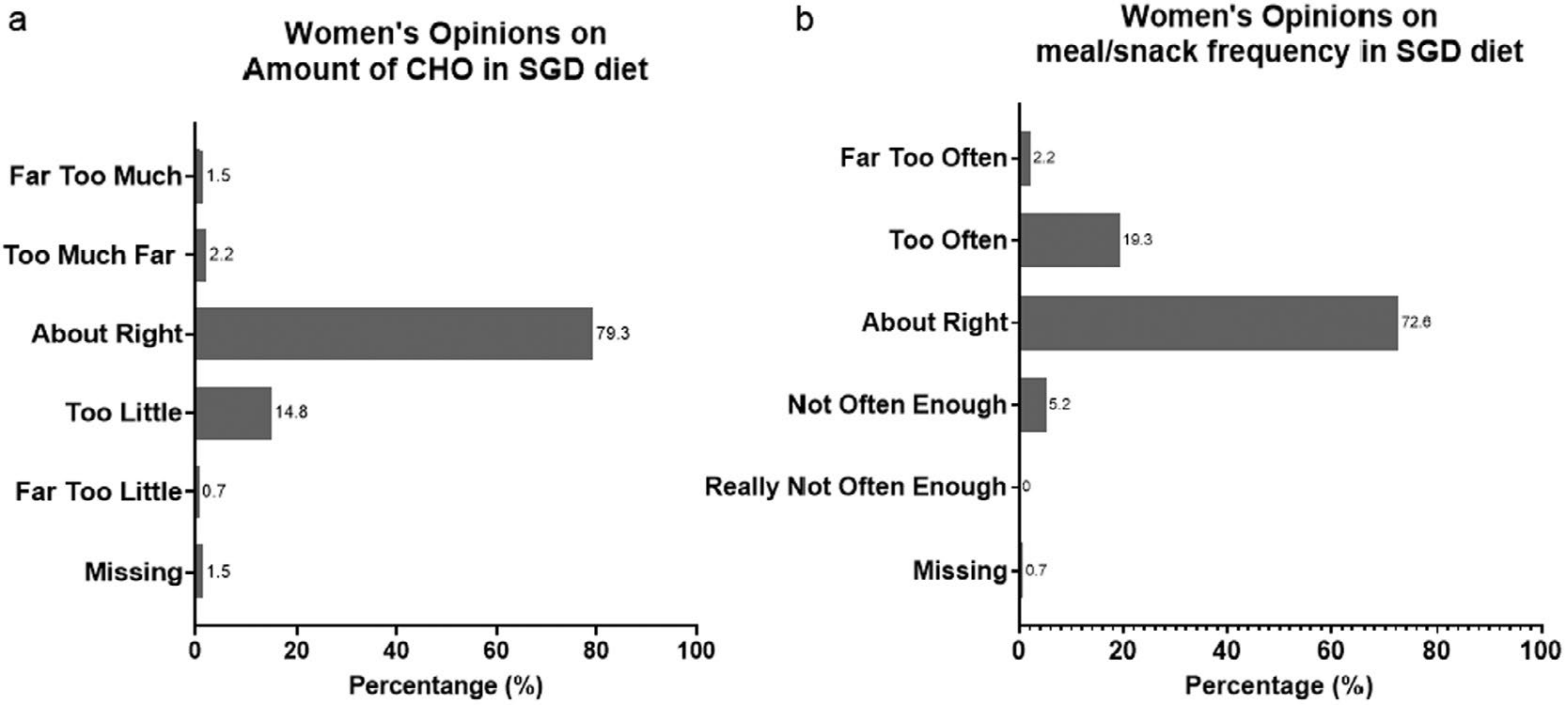
Women's opinion on SGD with regards to (a) Amount of Carbohydrates and (b) Meal/Snack frequency.

**FIGURE 2:**
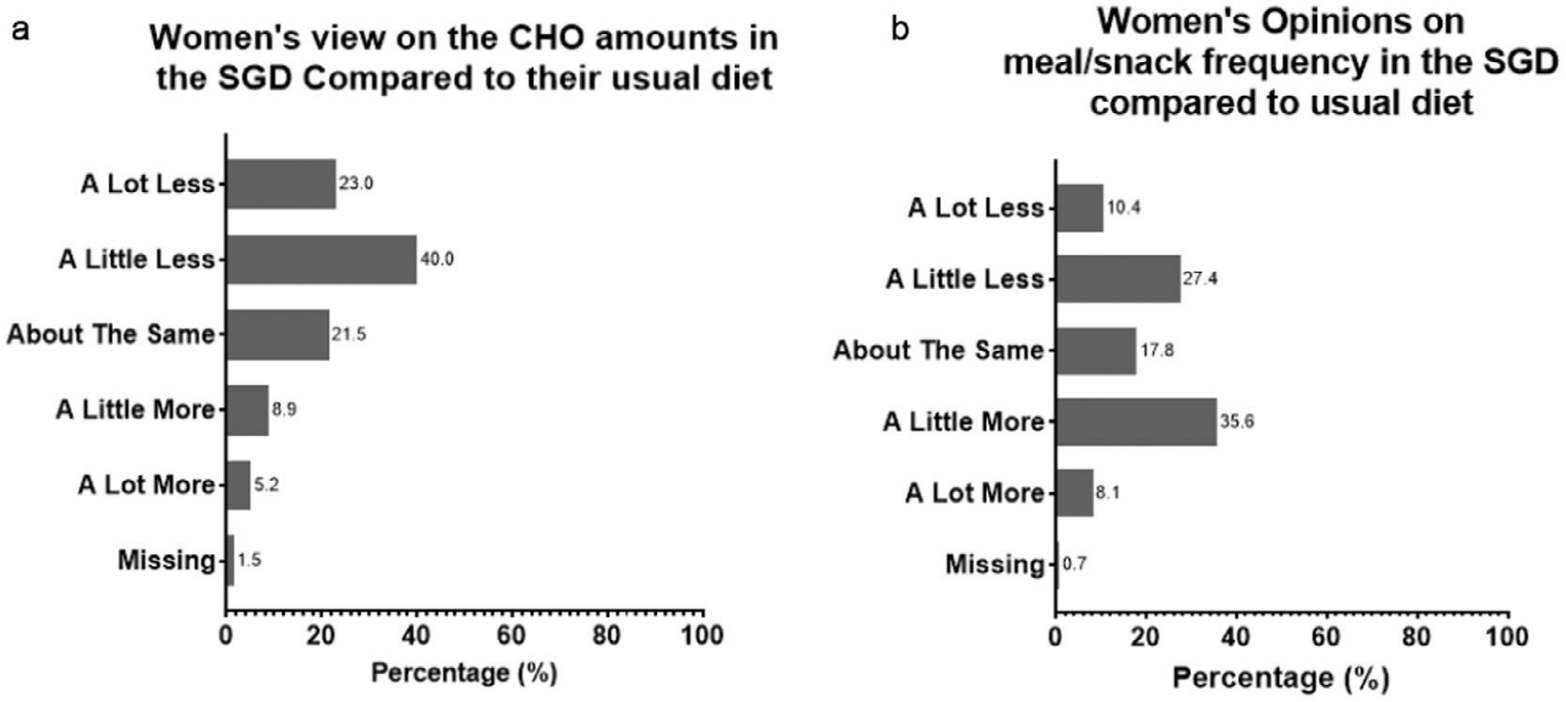
Women's opinion on SGD compared to usual diet—with regards to (a) Amount of Carbohydrates and (b) Meal/Snack frequency.

Of the 135 women surveyed (Table 1), most perceived SGD diet carbohydrate amounts and distribution as ‘about right’ 79.3% (*n* = 107) and 72.6% (*n* = 98), respectively (Figure 1). Only 3.7% (*n* = 5) of women reported carbohydrate amounts as ‘too much or far too much’. Middle Eastern women were more likely to perceive SGD diet carbohydrate amounts as ‘too little/far too little’ compared to other ethnicities (30.0 vs. 11.4% *p* < 0.01). More women reported the meal‐snack frequency as ‘too often/far too often’ compared to ‘not often enough/really not often enough,’ 21.5% (*n* = 29) vs. 5.2% (*n* = 7), respectively (*p* < 0.001) (Figure 2). There were no differences in perception of SGD diet according to hunger levels, pre‐pregnancy Body Mass Index and non‐Middle Eastern ethnicities.

**TABLE 1:** Population demographics.

Parameter	Mean ± SD^a^ or *n*/total (%)
Participant characteristics*	
Maternal Age (years) *n* = 126	30.7 ± 5.2
Weeks' Gestation at time of survey (weeks) *n* = 130	29.4 ± 6.5
**Anthropometry**	
Pre‐pregnancy weight (kg) *n* = 128	74 ± 19.6
Weight at time of survey (kg) *n* = 129	82.2 ± 19.8
Height (m) *n* = 108	1.61 ± 0.08
Pre‐pregnancy Body Mass Index (kg/m^2^) *n* = 108	28.4 ± 7.1
Obese pre‐pregnancy *n* = 108	34 (25.2)
**Ethnicity**	
Middle Eastern	30/135 (22.2)
European/Caucasian	20/135 (14.8)
South Asian	43/135 (31.9)
South East Asian	22/135 (16.3)
Polynesian	9/135 (6.7)
African	3/135 (2.2)
Missing data	8/135 (5.9)
**Level of education**	
Below high school	5/135 (3.7)
High school	26/135 (19.3)
TAFE	21/135 (15.6)
University	77/135 (57.0)
Missing data	6/135 (4.4)

^a^ Data are Mean ± SD or *n* = (%).

Many women reported they were advised to reduce their carbohydrate intake (62.9%, *n* = 85) and increase meal‐snack frequency (43.7% *n* = 59) at GDM education. Furthermore, dietary changes reported post SGD diet education included smaller meal portions (*n* = 32, 23.7%), eating more often (*n* = 50, 37.0%), eating less carbohydrates/sugar (*n* = 34, 25.2%) and improving diet quality (*n* = 56, 41.5%). Eighty‐four women (62.2%) would prefer to have more carbohydrate rich foods, while 39 women (28.9%) would choose to eat less of the carbohydrate rich foods than recommended in the SGD diet.

Some women reported feeling ‘very or a little hungry’ when following the SGD diet (*n* = 51, 37.8%); however, more reported hunger levels as ‘about right’ (*n* = 63, 46.7%).

Women correctly identified most grain‐based carbohydrate foods (bread, rice, cake) as carbohydrate‐rich foods (90% *n* = 121). However, only 20.0% (*n* = 27) and 31.1% (*n* = 42) of women correctly identified fruit (apple) and dairy (milk) as carbohydrate‐rich food items, respectively.

Most women surveyed reported the SGD diet ‘made good sense’ (83%, *n* = 112, agree/strongly agree). They reported the diet was useful for preventing hyperglycaemia, met their pregnancy nutritional needs (85.9%, *n* = 116, useful/very useful), and was practical (81.5%, *n* = 110, often/always). The majority also reported that they followed the SGD diet well (70.4%, *n* = 95 often/always). Despite reporting such adherence, only 26.7% (*n* = 36) reported the SGD diet was ‘easy/very easy’ to follow. Almost half of the women perceived the SGD diet as ‘challenging/very challenging’ to follow (48.2%, *n* = 65). The most widely reported challenges included applying the recommended meal‐snack frequency (*n* = 13) and reducing sugar and other carbohydrate‐rich foods (*n* = 23). Only one woman of Middle Eastern ethnicity reported the SGD diet ‘does not fit with my cultural food’ as a challenge.

## 4 Discussion

This novel cross‐sectional study assessed the lived perspectives of a multiethnic cohort of women following the SGD diet for GDM management. To the authors’ knowledge, this is the largest study specifically assessing the perspectives of women regarding the diet recommended to them for GDM management. Overwhelmingly, women did not perceive the overall carbohydrate amounts advised as ‘too much’. To the contrary, most reported the carbohydrate amount as ‘about right’, with many reporting being advised to reduce their carbohydrate intake and to eat more often compared to their baseline diet.

There was a high rate of South Asian, South East Asian and Middle Eastern women, and a low rate of women of European background (15.7%). A lack of culturally specific dietary advice has been reported by several studies specifically on dietary management of GDM [15, 16, 17, 18]. To the contrary, only one participant perceived that the dietary advice provided was not culturally appropriate in this study. Furthermore, 81.5% of participants selected ‘often’ or ‘always’ to the statement ‘I feel the dietitian understands what is practical for me to eat’, possibly a reflection of the culturally specific meal plans provided at our facility—which were further tailored to individual preferences.

Evidence from this study suggests that our clinic population perspectives of the SGD do not appear to be significantly influenced by the public popularity of low carbohydrate diets [11]. This is evident in the fact that 79.3% of women in this study reported that the SGD diet amounts were ‘about right’, and a significant proportion (28.9%) reported that the dietitian advised them to eat less carbohydrate. Additionally, 63% reported that dietitians advised reducing the amounts they were eating overall. Furthermore, the fact that a significant proportion of the women reported feeling ‘very or a little hungry’ (37.8%) when following the diet warrants further investigation.

The SGD diet requirement that women follow a structured meal plan involving six occasions of eating spread over the day could be a challenge for women, especially from certain cultural backgrounds who traditionally eat less frequently. However, less than 10% described the eating frequency as challenging. This is in contrast with a qualitative study by Lawrence et al. [16] reporting that some women found it challenging to fit the three meals and snacks into their daily routine.

Pregnancy has been described as a teachable moment [19, 20], thought to motivate individuals to adopt risk‐reducing health behaviours. Carbohydrate identification was taught as an important skill for women living with GDM, as it assists in interpreting SMBG results in relation to their carbohydrate meal load. Unfortunately, approximately one‐third of participants did not identify fruit and dairy as carbohydrate‐rich food sources. Future education should emphasise these as carbohydrate sources—both in terms of positive nutritional benefits, but also potential impact on BGLs. Taken together, the women's perspectives on the carbohydrate amounts recommended need to be interpreted in the light of the fact that most women were only providing a view on the grain‐based carbohydrate‐rich amounts recommended.

The knowledge gained by the women in this study is however evident in the appropriate dietary changes reported. Eating less sugar and other carbohydrate‐rich foods, and improved diet quality were the most reported dietary changes post SGD diet education. Similar dietary changes have been reported in other studies [21, 22].

The self‐reported high compliance rate in this study (70.4%) was despite almost half of women perceiving the SGD diet overall as challenging or very challenging (48.2%). Other studies have reported that women self‐report high compliance to the GDM diet due to maternal motivation for neonatal care and positive health outcomes [23]. We do not have any dietary data in this study to confirm the self‐reported compliance rate.

Low carbohydrate diets have had surges in popularity globally over the last 20 years [11]. A recent national Australian survey of dietitians found that a significant proportion were recommending low carbohydrate diets to women with GDM [10]. Fortunately, there was no evidence that women in this study wanted to follow a low carbohydrate diet to manage their GDM, as they were encouraged to minimise processed high fat and high sugar carbohydrate rich foods, but to continue to eat nutrient dense healthy sources in amounts needed to meet the nutritional requirements of pregnancy.

This study has several strengths. The first is the significant sample size. Many studies investigating perspectives of the GDM diet are qualitative in nature with small sample sizes. GDM prevalence has a high prevalence among non‐Caucasian women; a further strength is representation across the four major ethnicity groups. An additional strength is the anonymous nature of this survey, reducing the likelihood of women providing positive feedback to avoid offending their treating team.

This study has several limitations. First is the lack of dietary intake data. The compliance rate in this study is entirely self‐reported and cannot be validated against any quantitative measure of intake (such as a food frequency questionnaire or diet history). A further significant limitation was that a large proportion of women in this cohort were tertiary educated (57%), potentially due to interpreter‐requiring women being excluded. As the clinic population is in a geographical area with high rates of socioeconomic disadvantage, it is likely that this sample is not representative of our GDM population overall. More highly educated women agreed to participate in the survey—which potentially reduces the transferability/generalisability of results across our clinic and other similar cohorts.

In conclusion, the SGD diet for GDM was well received overall and resulted in significant self‐reported dietary changes. Taken together, these results suggest that the dietary advice was adequately tailored to the individual, including cultural background. Further research is needed; however, to investigate the cultural acceptance and affordability of the SGD diet, given the high rates of GDM found in lower SES multicultural clinic populations. Further research is also needed to determine how best to support women on the SGD diet, given the high proportion of women who found the diet challenging to follow. Finally, despite low carbohydrate diets increasing in popularity, there was no evidence from this study to support the notion that women were resistant to the amounts of carbohydrate recommended.

## Conflicts of Interest

The authors declare no conflicts of interest.

## Supporting information


**Supporting Information:** Patient Experience—Standardised Gestational Diabetes Diet Questionnaire.

## Data Availability

The data that support the findings of this study are available on request from the corresponding author. The data are not publicly available due to privacy or ethical restrictions.
