# Supplementary material for: Do Dietitians Recommend Too Much Carbohydrate? A Cross‐Sectional Survey of Gestational Diabetes Mellitus Dietary Advice: A Patient Perspective
Source: Aust N Z J Obstet Gynaecol. 2026 Apr 6;66(2):e70121. doi: 10.1111/ajo.70121 (PMC13051523; doi:10.1111/ajo.70121)
Supplement: Supplementary file 1 — Supporting Information: Patient Experience—Standardised Gestational Diabetes Diet Questionnaire. [file AJO-66-0-s001.docx]

**Supplementary material**

**Patient Experience - Standardised Gestational Diabetes Diet Questionnaire**

We would like to ask you some questions regarding your experience with the diet we recommended to you for managing Gestational Diabetes. Your feedback will provide us with valuable information. Please **circle or write** your response to the questions below.

1. What do you think about the amount of carbohydrates the dietitian recommended you eat?

| Far too little | Too little | About right | Too much | Far too much | Unsure |
| --- | --- | --- | --- | --- | --- |

2. Compared to the amounts of carbohydrates I ate before attending the Diabetes Centre, the dietitian recommended that I now eat:

| A lot more | A little more | About the same | A little less | A lot less | Unsure |
| --- | --- | --- | --- | --- | --- |

3. What types of food would you want to eat more OR less of, if you had the option?

MORE­­­­ ________________________________________________________________________

LESS _______________________________________________________________________­­_

4. What do you think about how often the dietitian recommended you eat?

| Really not often enough | Not often enough | About right | Too often | Far too often | Unsure |
| --- | --- | --- | --- | --- | --- |

5. Compared to how often I ate before attending the Diabetes Centre, the dietitian recommended that I now eat:

| A lot more | A little more | About the same | A little less | A lot less | Unsure |
| --- | --- | --- | --- | --- | --- |

6. What changes have you made to your usual eating since seeing the dietitian?

__________________________________________________________________________________________________________________________________________________________________________________________________________________________________________

______________________________________________________________________________

7. When following the meal plan I felt:

| Very hungry | A little hungry | About right | A little full | Very full | Unsure |
| --- | --- | --- | --- | --- | --- |

8. Please tick all of the following foods that are carbohydrates:

| 🞏 Meat | 🞏 Milk | 🞏 Lettuce | 🞏 Bread | 🞏 Rice | 🞏 Apple | 🞏 Cake | 🞏 Cabbage |
| --- | --- | --- | --- | --- | --- | --- | --- |

***Please turn over the page…***

9. How much do you agree with the following sentence: “The diet recommended by the dietitian makes good sense.”?

| Strongly disagree | Disagree | Neutral | Agree | Strongly Agree | Unsure |
| --- | --- | --- | --- | --- | --- |

10. How useful do you think the dietary advice is for preventing high glucose levels and meeting the nutritional needs of pregnancy?

| No at all useful | Not useful | Somewhat useful | Useful | Very useful | Unsure |
| --- | --- | --- | --- | --- | --- |

11. I feel that the dietitian understands what is practical for me to eat.

| Never | Rarely | Sometimes | Often | Always | Unsure |
| --- | --- | --- | --- | --- | --- |

12. I follow the meal plan recommended by the dietitian:

| Never | Rarely | Sometimes | Often | Always | Unsure |
| --- | --- | --- | --- | --- | --- |

13. I feel that following the GDM diet was:

| Very challenging | Challenging | Neutral | Easy | Very easy | Unsure |
| --- | --- | --- | --- | --- | --- |

If you found it ‘very challenging’ or ‘challenging’ please let us know why?

__________________________________________________________________________________________________________________________________________________________________________________________________________________________________________

14. Do you have any suggestions to improve the eating advice provided by the dietitian? ______________________________________________________________________________

____________________________________________________________________________________________________________________________________________________________

______________________________________________________________________________

Please complete the details below:

My age is­­­­­­­­­­­­­:­_______­­­­_______ How many weeks pregnant are you? __________________________

Education: Did Not Finish School 🞏 Finished High School 🞏 TAFE 🞏 University 🞏

Where were your parents born: _____________________________________________________

What was your weight before this pregnancy: ____________­______________________________

What is your current weight: __________________ What is your height: ____________________­­­

***Thank you for taking the time to complete this questionnaire***
